# Multiple cardiac surgical procedures in a case of an octogenarian with essential thrombocythemia

**DOI:** 10.1186/s40792-023-01682-z

**Published:** 2023-06-06

**Authors:** Motohiro Maeda, Shun-Ichiro Sakamoto, Tomohiro Murata, Atsushi Hiromoto, Kenji Suzuki, Yosuke Ishii

**Affiliations:** 1 Department of Cardiovascular Surgery, Nippon Medical School Musashikosugi Hospital, 1-396 Kosugi-Cho, Nakahara-Ku, Kawasaki-Shi, Kanagawa 211-8533 Japan; 2 Department of Cardiovascular Surgery, Nippon Medical School, 1-1-5, Sendagi, Bunkyo-Ku, Tokyo, 113-8603 Japan

**Keywords:** Essential thrombocythemia, Hydroxycarbamide, Hydroxyurea, Combined cardiac surgery, Perioperative management

## Abstract

**Background:**

Essential thrombocythemia (ET) is a chronic myeloproliferative disorder characterized by an elevation of platelet counts with a tendency for thrombosis and hemorrhage. The perioperative management of cardiovascular surgery of an ET patient is complicated. There is limited literature on the perioperative management of patients with ET undergoing cardiovascular surgery, particularly those requiring multiple procedures.

**Case presentation:**

An 85-year-old woman with a history of essential thrombocythemia (ET), which resulted in an abnormally high platelet count, was diagnosed with aortic valve stenosis, ischemic heart disease and paroxysmal atrial fibrillation. She underwent aortic valve replacement, coronary artery bypass grafting, and pulmonary vein isolation. The postoperative course was uneventful, nor hemorrhage and thrombosis.

**Conclusions:**

We represent a case of perioperative management and successful treatment of three combined cardiac surgery for an octogenarian ET patient who is the oldest case ever reported.

## Background

Essential thrombocythemia (ET) is a chronic myeloproliferative disorder characterized by an elevation of platelet counts with a tendency for thrombosis and hemorrhage. The perioperative management of cardiovascular surgery of an ET patient is complicated. First, cardiopulmonary bypass (CPB) management during surgery is complicated. Second, both thrombosis and hemorrhage are significant risks in the postoperative period of cardiac surgery, particularly those with uncontrolled thrombocytosis. Prior literatures about postoperative management of ET patients undergoing cardiovascular surgery are few and the reported operations consist of one or two surgical procedures. We represent a case of perioperative management and successful treatment of three combined cardiac surgeries for an octogenarian ET patient who is the oldest case ever reported.

## Case presentation

An 85-year-old woman was referred to our hospital for the surgical management of aortic valve stenosis, angina pectoris, and paroxysmal atrial fibrillation with New York Heart Association class III heart failure.

A decade ago, the patient was diagnosed with essential thrombocythemia (ET). She had consulted a hematologist at another facility and was treated with low-dose aspirin (100 mg/day) and hydroxycarbamide (hydroxyurea, HU) (500 mg/day).

Patient’s left ventricular ejection function reduced to 35%, as determined using transthoracic echography. The aortic valve was calcified and its area was 0.30 cm2; the peak pressure gradient across the aortic valve was 70 mmHg. Coronary angiography revealed triple vessel lesions involving critical stenosis of the right coronary artery (RCA #2; 90%) and the left anterior descending coronary artery (LAD #7; 75%). The patient was also diagnosed with chronic kidney disease and peripheral arterial disease.

Preoperatively, the low-dose aspirin was replaced by heparin, which increased the activated partial thromboplastin time from 40 to 50 s. A known side effect of HU is delayed wound healing. Therefore, as her hematologist suggested, we planned to maintain the platelet count at < 100,000/µL and discontinued HU 1 day before surgery. However, 3 days preoperatively, HU had to be stopped, because the patient’s platelet count markedly decreased from 300,000 to 87,000/µL, following which, the platelet count increased to 150,000/µL just before surgery.

The surgical procedure involved aortic valve replacement, coronary artery bypass grafting (CABG), and pulmonary vein isolation (PVI). The aortic valve was replaced with a 19 mm bioprosthetic prosthesis (Carpentier Edwards Perimount Magna Ease, Edwards Life science, Irvine, CA, US). The bypass design was from the left internal mammary artery to the LAD and from the saphenous vein (SV) to the RCA. CABG and PVI were performed on pump beating. Proximal anastomosis of the SV was achieved using the HEARTSTRING III Proximal Sealing System (MAQUET Holding B.V. & Co. KG, Rastatt, Germany). Activated clotting time was maintained over 450 s during cardiopulmonary bypass (CPB). The reservoir did not need to be changed, and no clots were captured during CPB. The CPB, aorta clamping, and operation times were, respectively, 178 min, 88 min, and 384 min. Intraoperative blood transfusion included 2240 mL of erythrocytes, 840 mL of fresh frozen plasma, and 500 mL of platelets.

**Fig. 1:**
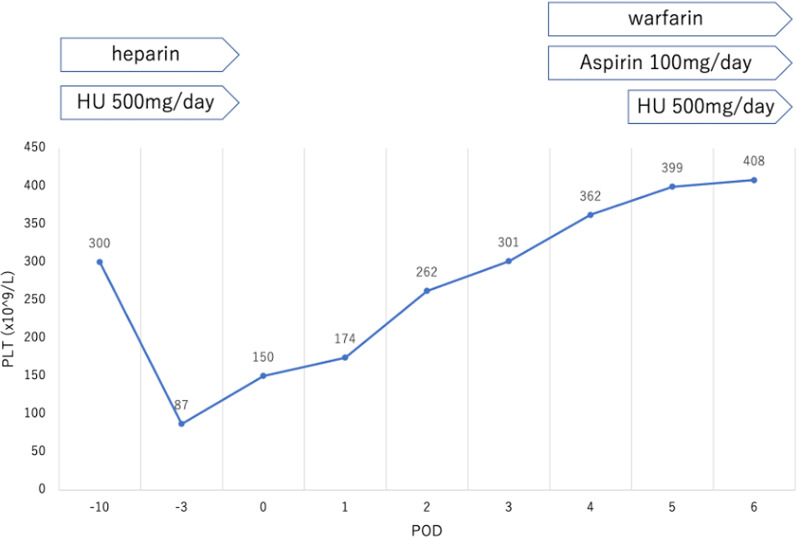
Preoperative and postoperative platelet count with the treatment course. *HU* hydroxyurea, *PLT* platelet, *POD* postoperative day

Postoperatively, low-dose aspirin (100 mg/day) was resumed as little or no postoperative bleeding was observed, and coumadin was initiated as anticoagulant therapy for valve replacement. Four days after the surgery, the platelet count increased enough for HU to be resumed at the same dose as that preoperatively (Fig. 1).

**Fig. 2:**
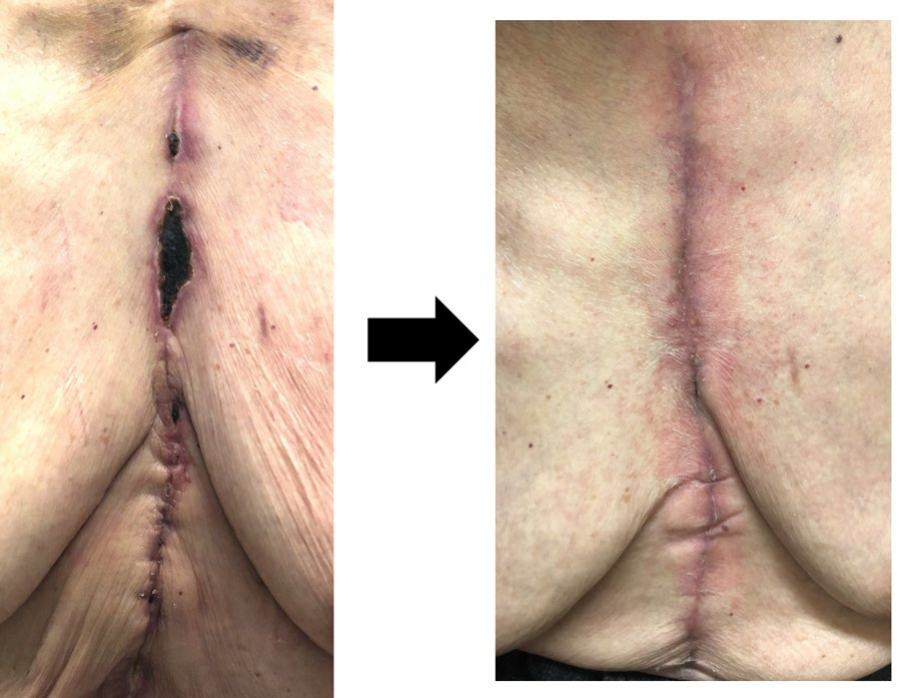
Surgical wound of median sternotomy

During treatment, the patient experienced no complications, such as hemorrhage or thrombosis. However, prolonged hospitalization was required for protracted wound healing, a side effect of HU (Fig. 2).

The patient did not experience heart failure throughout the postoperative course and was discharged from the hospital on the 22nd day postoperatively.

## Discussion

ET is a rare myeloproliferative disorder that is characterized by excessive proliferation of persistent thrombocytosis (≥ 450 × 10^9^/L). According to the World Health Organization diagnostic criteria, bone marrow biopsy shows an increase in enlarged and mature megakaryocytes [1]. Patients with ET are at a high risk of thrombotic and bleeding complications. Age > 60 years and a previous thrombotic event are particular risk factors for thrombosis. Furthermore, a platelet count of > 1500 × 10^9^/L is a risk factor for bleeding [2]. Patients with ET present with platelet dysfunction with a decrease in von Willebrand ristocetin cofactor activity and high molecular weight von Willebrand factor multimers [3]. This phenomenon prolongs the bleeding time, whereas the prothrombin time and activated partial thromboplastin time are usually within reference ranges. Prophylactic anticoagulation for patients with ET before surgery may also contribute to postoperative bleeding complications.

Particularly, cardiac surgery puts patients with ET at a high risk of hemostatic complications. One reason for this is that most cardiac surgeries are performed through CPB. CPB has a unique feature of widespread activation of the hemostatic system. Platelet activation and aggregation is influenced upon exposure to the CPB component. Heparinization must be strictly administered during CPB. Activated clotting time is maintained at more than 450 s during CPB at our institute. Manaka reported the case of a patient with ET who underwent vascular surgery with heparin residence [4]. In such cases, argatroban is recommended instead of heparin. Another reason may be that the postoperative hemostatic system alters coagulation activation, platelet activation, and fibrinolysis [5]. As mentioned above, this tendency is mainly derived from blood contact with the CPB circuit and blood suction from the operative field. Furthermore, hypothermia and hemodilution contribute to hemostatic degeneration. In our patient, the rectal temperature during the surgery remained normothermia at approximately 36.0 °C. Another reason could be that in many cardiac surgeries, antiplatelet agents or anticoagulants must be initiated for postoperative treatment. The Japanese guidelines recommend anticoagulant therapy with warfarin for the first 3–6 months after bioprosthetic valve implantation as Class IIa [6]. We followed the guidelines for our patient; prothrombin time and international normalized ratio was controlled between 2.00 and 2.50.

Some case reports have described the perioperative course of patients with ET undergoing cardiac surgery. Previous studies on the postoperative management of patients with ET undergoing cardiovascular surgery are few. While most reports focused on the sole procedure, some have described combined cardiac surgery comprising two procedures (Table 1) [7–9]. In our patient, the three cardiac procedures were performed simultaneously. Moreover, ours was the oldest patient with ET reported to have undergone cardiac surgery.

**Table 1:** Reported combined cardiac surgeries with two procedures for patients with essential thrombocythemia

Author(s)	Age	Sex	Operation	Pre-OP therapy	Post-OP therapy	Complication/death
Gurrieri C et al., 2018	73	F	AVR, CABG × 1	Aspirin, warfarin	Heparin, warfarin	None
	74	F	AVR, MVP	Aspirin	Aspirin	Death (POD 20)
	80	F	Redo-AVR, CABG × 1	Aspirin, hydroxyurea	Aspirin, hydroxyurea	None
	60	F	AVR, myectomy	Aspirin	Aspirin, warfarin	None
	77	M	AVR, CABG × 2	Aspirin, hydroxyurea	Aspirin, hydroxyurea	None
	83	M	AVR, CABG × 1	Clopidogrel, hydroxyurea	Clopidogrel, hydroxyurea, warfarin	None
Kawaguchi Y et al., 2020	80	F	MVP, TAP	Hydroxyurea	Aspirin, hydroxyurea, warfarin	None
Suzuki K et al., 2020	70	F	Dsc Ao Replacement, Bowel resection	Aspirin	Heparin	Death (POD 56)

*AVR* aortic valve replacement, *CABG*  coronary artery bypass grafting, *MVP*  mitral valve plasty, *TAP* tricuspid valve annuloplasty, *Dsc Ao*  descending aorta, *Pre-OP* preoperative, *Post-OP*  postoperative, *POD*  postoperative day

Considering that long CPB is a risk factor for clotting complications, particularly for patients with ET, some strategies must be adopted to shorten CPB time. The patient in our case underwent on-pump beating CABG, because the preoperative low cardiac function was a risk in off-pump CABG. However, off-pump CABG is preferred over on-pump CABG given the platelet-related complications associated with on-pump CABG. An alternative treatment strategy is feasible including PCI or OPCAB, TAVI, and transcatheter PV isolation. The transcatheter cardiac intervention may provide benefits to the patient with special characteristics, such as old age, frailty, and complication. On the other hand, multiple cardiovascular pathologies tend to require a staged-hybrid procedure. In this case, we do the one-time surgical procedure of AVR, CABG, and PV isolation after a discussion with the cardiologist. It is still controversial whether to perform TAVI for patients with coronary artery disease [10, 11]. In addition, the surgical ablation of atrial fibrillation with AVR or CABG is not a perioperative risk, compared with lone AVR or CABG [12]. PV isolation has the advantage of a concomitant left atrial appendage resection, though the transcatheter ablation is incapable of the procedure. Prolonged CPB time is only the judgment for conversion of the surgical procedures. In this situation, omission of CABG or reduction of the number of anastomoses was considered, while CPB time in our case was acceptable.

The patient in this case report had no major postoperative complications except for wound healing delay. HU is a cytoreductive agent used to treat chronic myeloproliferative disorders. There is an association between HU and painful ulcers, particularly in the malleolar area. The inhibition of DNA synthesis, macrocytosis, and platelet dysregulation induced by HU therapy influences keratinocyte dysfunction, microthrombus formation, and impaired tissue repair [10]. This mechanism may also cause surgical wound coaptation to delay.

## Conclusions

We performed combined cardiac surgery with three procedures for an octogenarian patient with ET; this is the oldest case agewise to be reported. Careful and attentive perioperative and postoperative management leads to an adequately acceptable postoperative course.

## Data Availability

Not applicable.

## Abbreviations

ET: Essential thrombocythemia
AVR: Aortic valve replacement
CABG: Coronary artery bypass grafting
HU: Hydroxyurea
PVI: Pulmonary vein isolation
LIMA: Left internal mammary artery
LAD: Left anterior descending
SV: Saphenous vein
RCA: Right coronary artery
CPB: Cardiopulmonary bypass
ACT: Activated clotting time
